# Editorial Comment: Does the Use of a Robot Decrease the Complication Rate Adherent to Radical Cystectomy? A Systematic Review and Meta-Analysis of Studies Comparing Open with Robotic Counterparts

**DOI:** 10.1590/S1677-5538.IBJU.2020.04.06

**Published:** 2020-05-20

**Authors:** Eliney F. Faria

**Affiliations:** 1 Serviço de Urologia Hospital Felicio Rocho Belo HorizonteMG Brasil Serviço de Urologia, Hospital Felicio Rocho, Belo Horizonte, MG, Brasil

Tzelves L^1^, Skolarikos A ^1^, Mourmouris P ^1^, Lazarou L ^1^, Kostakopoulos N ^1^, Manatakis DK^2^, Kural AR ^3^

^1^2nd Department of Urology, Sismanoglio General Hospital, National and Kapodistrian University of Athens, Athens, Greece; ^2^ 1st Department of Surgical Oncology, St. Savvas Cancer Hospital, Athens, Greece; ^3^ Department of Urology, School of Medicine, Acibadem Maslak Hospital, Acibadem Mehmet Ali Aydinlar University, Istanbul, Turkey

J Endourol. 2019 Dec;33(12):971-984.

**DOI: 10.1089/end.2019.0226 | ACCESS: 10.1089/end.2019.0226**

## COMMENT

In this paper the group of university of Athens, performed a very good review and meta-analysis (using PRISMA guidelines) about complication rates of robotic assisted radical cystectomy (RARC). Despite open radical cystectomy (ORC) remains the mainstay of treatment for muscle-invasive and high-risk nonmuscle-invasive bladder cancer decreasing complication rates was the main goal of development of minimally invasive alternative techniques. RARC has been transforming into a safe and efficient alternative to the open gold standard procedure ( 1 - 3 ). This meta-analysis is the largest in the literature comparing complication rates between open and RARC. The advantages in terms of peri- and postoperative outcomes of this minimally invasive procedure has remained contradictory. A higher level of evidence is usually extracted by well-designed, randomized control studies and seems to agree with their findings that do not award the robotic procedure any advantage in terms of complication rates when compared with its open counterpart ( 3 - 6 ). They analyzed 54 studies (5 randomized trials and 49 observational), including 29,697 patients (6,500 in the RARC group and 23,197 in the open radical cystectomy group). RARC was associated with lower blood transfusion rates (p < 0.001), lower length of stay (p < 0.001), faster return to regular diet (p < 0.001), and lower postoperative mortality rates (p < 0.001), but longer operating time. They concluded RARC appears to be associated with fewer complications and favoring perioperative outcomes in comparison with the ORC. RARC is an efficient and safe procedure that can provide an alternative to the open procedure.
